# Bovine brucellosis in Pakistan; an analysis of engagement with risk factors in smallholder farmer settings

**DOI:** 10.1002/vms3.165

**Published:** 2019-04-08

**Authors:** Shumaila Arif, Peter C. Thomson, Marta Hernandez‐Jover, David M. McGill, Hassan M. Warriach, Khizar Hayat, Jane Heller

**Affiliations:** ^1^ School of Animal and Veterinary Sciences Charles Sturt University Wagga Wagga New South Wales Australia; ^2^ Graham Centre for Agricultural Innovation Charles Sturt University Wagga Wagga New South Wales Australia; ^3^ School of Life and Environmental Sciences The University of Sydney Camden New South Wales Australia; ^4^ Faculty of Veterinary and Agricultural Sciences The University of Melbourne Melbourne Victoria Australia; ^5^ AVCCR Dairy‐Beef Project University of Veterinary & Animal Sciences Lahore Pakistan

**Keywords:** bovine brucellosis, Pakistan, risk factors, seroprevalence, smallholder farmers

## Abstract

A cross‐sectional study was conducted to estimate the seroprevalence of bovine brucellosis in smallholder farms in seven regions in Pakistan, identify herd and individual level risk factors for seropositivity and assess the level of engagement of farmers with risk factors. In total, 1063 cattle and buffalo belonging to 420 herds in seven districts were sampled. The Rose Bengal test (RBT), indirect enzyme‐linked immunosorbent assay (I‐ELISA) and competitive enzyme‐linked immunosorbent assay (C‐ELISA) were used for the serological diagnosis of bovine brucellosis on all the serum samples. The associations between herd‐and animal‐level risk factors and seropositivity were investigated using logistic regression analyses. In addition, herd management practice scores, created to quantify the number of management practices undertaken that pose a risk for *Brucella* transmission, were calculated and compared between seropositive and negative herds within each district. Overall herd and animal prevalence were estimated to be 16.2% (95% CI, 13–20%) and 8.7% (95% CI, 7.2–10.6%), respectively, across all districts sampled. Herds with a history of last trimester abortion were found to be more likely to be positive than herds without such history (OR = 2.06, 95% CI, 1.09–3.89), providing validation of our findings and identifying that clinical disease is occurring in this region. It was also identified that herds with five to eight buffalo (OR = 3.80, 95% CI, 1.69–8.49), and those with more than eight buffalo (OR = 3.81, 95% CI, 1.51–9.58) were more likely to be positive for *Brucella* than those with less (one to two and three to four) buffaloes present in the herd. The presence of other domestic animals at the farm and purchasing animals in last year were found to have no association with seropositivity. The findings of this study support the need for the development of targeted intervention strategies specific to the disease status of each district.

## Introduction

Brucellosis is a highly zoonotic bacterial disease affecting humans and livestock worldwide (Pappas *et al*. 2006). The Food and Agriculture Organization (FAO), World Health Organization (WHO) and World Organisation for Animal health (OIE) consider brucellosis as one of the most widespread zoonoses in the world (Schelling *et al*. 2003).

Brucellosis is caused by different species of the genus *Brucella*. The major species of *Brucella* and their hosts are *B. abortus* (cattle), *B. melitensis* (goats), *B. suis* (pigs) and *B. ovis* (sheep). In humans, brucellosis can be caused by *B. abortus*,* B. melitensis* and *B. suis*. In livestock, brucellosis mostly affects sexually mature animals and may cause last trimester abortion storms during the breeding season. Additional clinical signs include retained placenta, repeat breeding syndrome, metritis, placentitis and weak calves and bulls may develop infertility due to epididymitis (Shareef 2006). Infected animals shed bacteria in aborted foetal material, uterine discharge, vaginal secretions and milk (England *et al*. 2004). The pathogen may be transmitted to other animals through ingestion of contaminated material, wounds or intact skin and mucous membranes (Bercovich 1998; Kahn *et al*. 2010). Infected bulls shed bacteria in their semen during the acute phase of the disease (McCaughey & Purcell 1973). However, pathogen transmission through breeding depends on the breeding method. Venereal transmission by natural breeding remains uncommon but using the semen from an infected bull for artificial insemination is possible source for the spread of the disease (Rankin 1965).

The epidemiology of bovine brucellosis is complex and influenced by different factors. These factors have been classified as animal population characteristics, management practices and the biology of the disease (Silva *et al*. 2000; Amin *et al*. 2005). Bovine brucellosis has been associated with a number of animal‐level risk factors including age, breed, body condition score and gender. Similarly, herd‐level risk factors for the disease include abortion history, herd size, insemination method and farm management practices (including lack of disinfection of environment after abortion, sharing calving space, new animal purchases and common grazing with animals from different herds) (Makita *et al*. 2011; Anka *et al*. 2014; Lindahl *et al*. 2014). All these factors and practices contribute either to contaminate the environment or act as a source for pathogen transmission. The ability of *Brucella* to survive in humid and cold environments for long periods of time is also an important factor for defining the risk of *Brucella* transmission to both animals and humans (Aune *et al*. 2012), with climatic variables playing a significant role in the epidemiology of the disease in different geographical zones.

Bovine brucellosis has been eradicated in many developed countries, including Australia, Canada, Israel, Japan, New Zealand and parts of Europe (Khan 2007). However, It remains an uncontrolled problem and endemic in areas such as Africa, the Mediterranean, the Middle East and in parts of Asia and Latin America (Refai 2002). The World Animal Health Information System (WAHIS), maintained by the OIE, states there are many clinical cases in the Middle East, Africa and Latin America but no data are available for Pakistan (WAHIS Interface OIE, 2016). In Pakistan, the dairy industry plays a pivotal role in the country's economy and is also a major source of income for rural communities. The industry is predominantly represented by smallholder farms, with 90% of the country's dairy industry based on farms with less than 10 animals (Afzal 2009). Cattle and water buffalo are the major milk‐producing animals, and have an estimated population of 38 million and 44 million, with an annual production of 20 143 and 34 122 million tons of milk, respectively (Pakistan Economic Survey, 2016). The country can been divided into ten agro‐ecological zones based on the climate, water availability, land use, resources and geography (Khan 2004). Pakistan is located in a subtropical zone with a humid climate in some regions (Farooqi *et al*. 2005). This could be a favourable environment for *Brucella* survival and spread if disease is present (Aune *et al*. 2012). A recent study has confirmed the presence of *Brucella abortus biovar 1* using polymerase chain reaction (PCR) in cattle in Pakistan (Ali *et al*. 2014). Munir *et al*. (2011) has reported the seroprevalence of *Brucella abortus* in buffalo and cattle is 15.2% and 9%, respectively, on organised large farms and government farms using an indirect ELISA method. However, most of the reported studies were carried out on large commercial mixed dairy farms or research stations, which represent a small fraction (only 10%) of the industry. The available prevalence estimates of bovine brucellosis are not generalisable for the smallholder production system as herd management practices and disease exposures are entirely different for this system. In addition, smallholder farming systems are associated with people that frequently have a low level of education, lack of biosecurity knowledge and practice and high prevalence of practices which pose risk for zoonotic diseases transmission (Arif *et al*. 2017). All these factors indicate that there is greater chance of spreading disease both to animals and humans if the disease is present in the system on smallholder farms than on larger commercial dairies.

This study aimed to estimate the seroprevalence and associated risk factors (at both the animal and herd level) of bovine brucellosis in mixed buffalo and cattle smallholder farms in seven districts in Pakistan. It is expected that the outcomes of this study will help to devise an intervention according to the disease status of districts through an educational awareness or extension programme.

## Materials and methods

The study was conducted in collaboration with ASLP (Agriculture Sector Linkages Program) dairy extension research Project (LPS/2010/2007) through the Australian Centre for International Agriculture Research (ASLP dairy project, 2010). Between February and June 2015 a cross‐sectional study was carried out to estimate the herd‐ and animal‐level prevalence of bovine brucellosis in smallholder farming systems of Pakistan. This study was conducted in five districts of Punjab (Okara, Pakpattan, Kasur, Jhelum and Bhakkar) and two districts of Sindh (Badin and Thatta). Sampling strategies including districts, villages and farms have been described in detail by Arif *et al*. (2017). In short, two villages per district and 60 herds per village were sampled. The demographic characteristics of the selected districts are presented in Table 1.

**Table 1:** Demographic features of study districts of Pakistan. Source: (Pakistan Bureau of Statistics 2016)

Province	District	Ago‐ecological zone	Agro‐climatic zone	Number of buffalo per km^2^ area	Number of cattle per km^2^ area	Human population
Punjab	Kasur	Northern irrigated	Arid	271.46	97.64	2 375 875
	Okara	Northern irrigated	Arid	201.13	75.26	2 232 992
	Pakpattan	Northern irrigated	Arid	213.29	70.34	1 286 680
	Jhelum	Barani land	Arid	40.63	46.49	936 957
	Bhakkar	Sandy dessert	Hyper arid	31.98	66.02	1 051 456
Sindh	Thatta	Indus delta	Hyper arid	54.58	61.04	1 136 044
	Badin	Indus delta	Hyper arid	28.77	18.17	1 113 194

Sera from cattle (*n *=* *441) and buffalo (*n *=* *621) were collected from smallholder mixed cattle and buffalo dairy farms. In total, 420 farms were selected and a maximum of three animals (given the availability of animals on individual farms) were randomly sampled from each farm (so that a sizeable proportion of each herd was sampled). No study farms vaccinated their cattle or buffalo against bovine brucellosis. The sample size was calculated based on an unknown disease prevalence (thus assumed to be 50%) at a herd level, a 95% confidence interval (CI) based on a normal distribution approximation, and a desired absolute precision of 5%. The above sampling approach was also appropriate for animal‐level prevalence assuming unknown prevalence. Epitools, an online epidemiological calculator (Sergeant 2017), was used for sample size calculation.

Oral consent was obtained from farmers prior to the start of the study and a pre‐designed questionnaire was used to collect information on herd‐ and animal‐level risk factors. The family member responsible for daily herd management was interviewed in Urdu, Punjabi, or Sindhi language, depending on farmer's native language. Location coordinates (latitude, longitude and altitude) of each farm were also recorded using iSURVEY™ application (https://www.harvestyourdata.com/). Climate data regarding maximum and minimum temperature, humidity and rainfall were obtained from local weather station of each district for the available years. Climate data for district Jhelum were available for the years 2010–15, Bhakkar: 2010–14; Kasur: 2014; Okara: 2010–14; Thatta: 2011–15 and Badin: 2011–15. No climate data were available for Pakpattan as there is no weather station in the district.

### Serum collection

A total of 1063 blood samples were collected from the jugular vein of each animal aseptically, according to the procedure described by Alton *et al*. (1975). Samples were kept cold at 4°C during transport to the laboratory at the University of Veterinary & Animal Sciences, Lahore Pakistan. Commercially available serum separator vacutainers were used for serum collection. Serum was stored at −20°C until diagnostic tests were performed in the same laboratory.

### Diagnostic tests

Three serological tests (RBT, I‐ELISA and C‐ELISA) were performed on all sera collected from the seven districts.

### Rose Bengal test (RBT)

The RBT was performed as recommended by the OIE (World Organisation for Animal Health, 2009). Briefly, 30 *μ*l of each serum sample was mixed thoroughly using a clean glass rod for each sample with 30 *μ*l of RBT antigen on the clean transparent slide. The mixture was agitated softly for 4 min at 22°C. The reaction was immediately assessed after 4 min as positive if agglutination was observed and negative if there was no reaction between serum and antigen. The antigen, positive and negative controls were procured from Institute Pourquier Rose Bengal Ag by IDEXXTM.

### Indirect enzyme‐linked immunosorbent assay (I‐ELISA)

The I‐ELISA was performed using a commercial kit (IDEXX Brucellosis Serum X2 Ab Test) procured from IDEXXTM Laboratories, USA. The test was performed according to the recommendation of the manufacturer. Results are expressed as the ratio of the sample optical density (OD) minus the mean kit negative control OD to the mean kit positive control OD minus the mean kit negative control OD (S/P ratio). A positive result was defined by the manufacturer as an S/P ratio of ≥80%, and negative for an S/P ratio of <80% (http://www.idexx.com.au/livestock-poultry/ruminant/b-abortus.html).

### Competitive enzyme‐linked immunosorbent assay (C‐ELISA)

The C‐ELISA was performed with SvanovirTM Brucella‐Ab C‐ELISA kit procured from Svanova Biotech, Uppsala Sweden using the procedure described by World Organisation for Animal Health (2009) and Matope *et al*. (2011). Percent inhibition (PI) was calculated to measure antibody titres using the formula suggested by the manufacturer of the kits:

$$\text{PI}=100-\frac{\text{Mean OD value of sample or control}}{\text{Mean OD value of conjugate control}}\times 100$$

### Statistical analysis

A number of samples returned negative results for RBT and positive for ELISA, which is inconsistent with what was to be expected with the use of RBT as screening and ELISA as confirmatory tests. Therefore, a Bayesian latent class approach was used to evaluate three diagnostic tests simultaneously for field conditions in Pakistan. The result from the evaluation study suggests using RBT and C‐ELISA in parallel combination produced the highest overall sensitivity and specificity (Arif *et al*. 2018), when considered as their sum. Consequently, this definition was adopted in this study. A herd that had at least one positive animal in either of these two tests was classified as a positive herd. Initial infographics were plotted using descriptive statistics to show the pattern of brucellosis prevalence in both sexes of buffalo and cattle, within each district. A spatial distribution map showing herd location and *Brucella* seropositive herd status was constructed using ArcGIS^®^ and ArcMap™. Climate data were plotted using averages of each variable, i.e. maximum and minimum temperature, humidity and rainfall over 12 months for the given years of each district. Note that district Pakpattan was excluded from the graphs, although is adjacent to Okara with similar altitude and landscape, and presumably has a similar climate. Climate graphs were plotted using the lattice package with R (Sarkar 2008). This was followed by logistic regression modelling to assess factors associated with disease presence. Districts Bhakkar, Thatta and Badin had no positive farms for brucellosis so the data of these districts were removed from the logistic regression models. The response variable for (the outcome of RBT and C‐ELISA) were coded as 1 (positive) vs. 0 (negative). Herd‐level risk factors used as explanatory variables in univariable logistic regression models were (1) number of buffalo; (2) number of cattle; (3) retained placenta cases in the last year (Yes vs. No); (4) last trimester abortions (Yes vs. No); (5) presence of other domestic animals at farm (Yes vs. No) and (6) animals purchased in last 1 year (Yes vs. No). Similarly, animal‐level risk factors were also screened with univariable logistic regression: (1) age; (2) species (cattle vs. buffalo) and (3) body condition score. Animal sex was excluded from the univariable analysis as only a few bulls were sampled. Explanatory variables with *P *<* *0.20 in the univariable analysis were considered for further assessment in the multivariable analysis. A backward elimination procedure was used to build the final multivariable model with the inclusion criteria set was *P *<* *0.05. Models were fitted using the *glm()* function in R (R core Team, 2015).

In addition, two scores were calculated. These were the total number of risky herd management practices undertaken by farmers in two separate categories, i.e. a farm cleaning risk score and a brucellosis herd transmission risk score, as described in a previous study on the same farmers (Arif *et al*. 2017). Briefly, the farm cleaning risk score (scored from 0 to 4) was the total number of risky practices practised by the farmer of the following four herd management practices: (1) not cleaning up dung; (2) not cleaning the feeding trough; (3) storage of dung piles for more than 6 months and, (4) not washing udder before milking. Brucellosis herd transmission risk score (scored from 0 to 5) was based the following risky herd management practices: (1) common grazing for animals; (2) not disinfecting space after birth; (3) not disposing of placental membranes; (4) calving space shared with other animals and, (5) slaughter of animals on‐farm. These two indexes were plotted, using bar graphs, to visualise the distribution of scores both for seropositive and negative herds for the seven districts.

## Results

A total of 420 smallholder dairy farms were involved in this study, and sera were obtained from 441 cattle and 621 buffalo to estimate seroprevalence and associated risk factors of brucellosis in Pakistan. Of the 420 farms, two farms were removed because restraint of the animals was not possible at the farm location. In addition, five farms were removed from the spatial distribution map of bovine brucellosis due to missing information regarding location coordinates.

### Seroprevalence of *Brucella*

**Figure 1:**
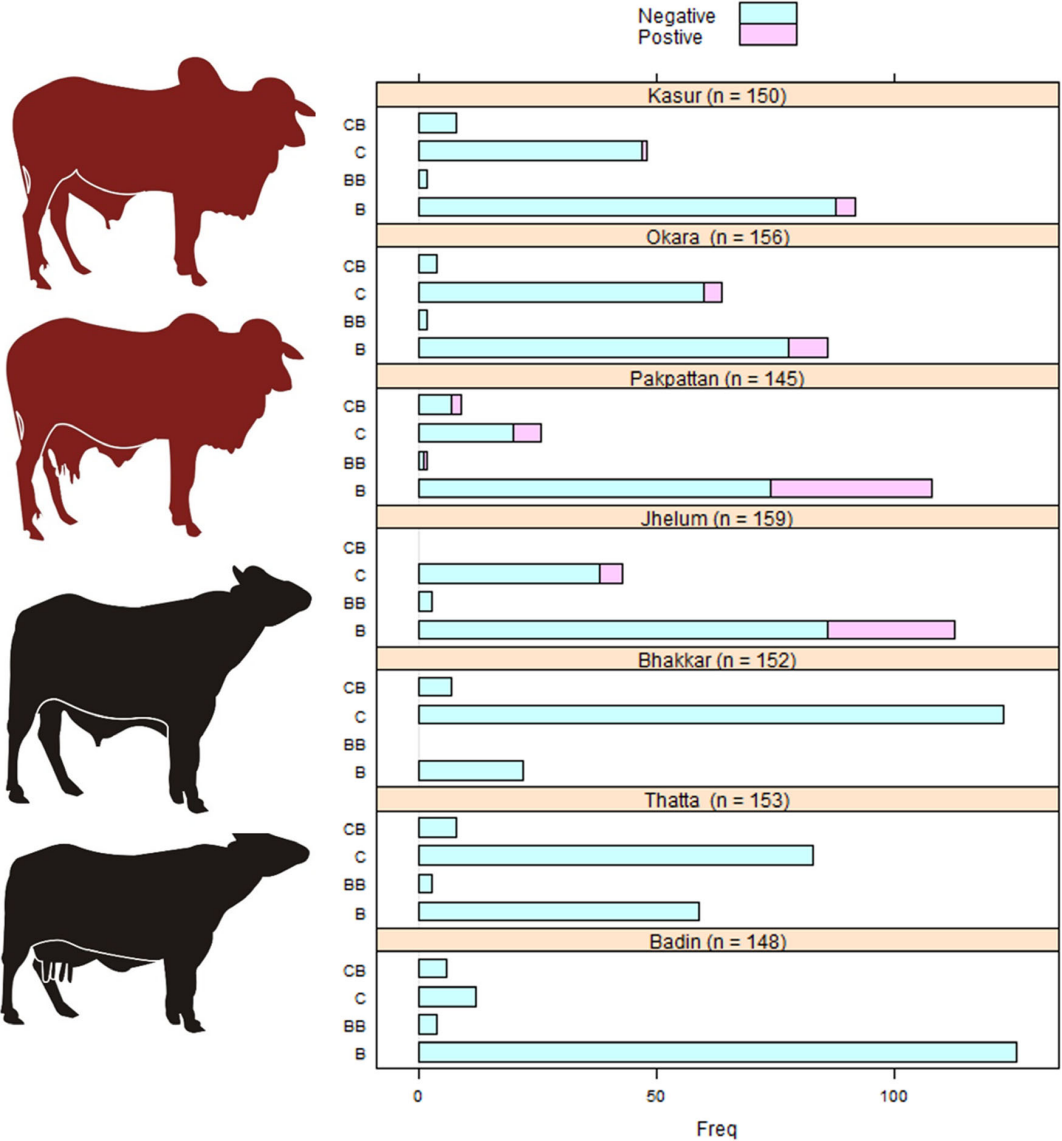
Infographic indicating the number of buffalo (B), buffalo bull (BB), cattle (C) and cattle bull (CB) along with animal‐level prevalence within individual species and districts.

**Figure 2:**
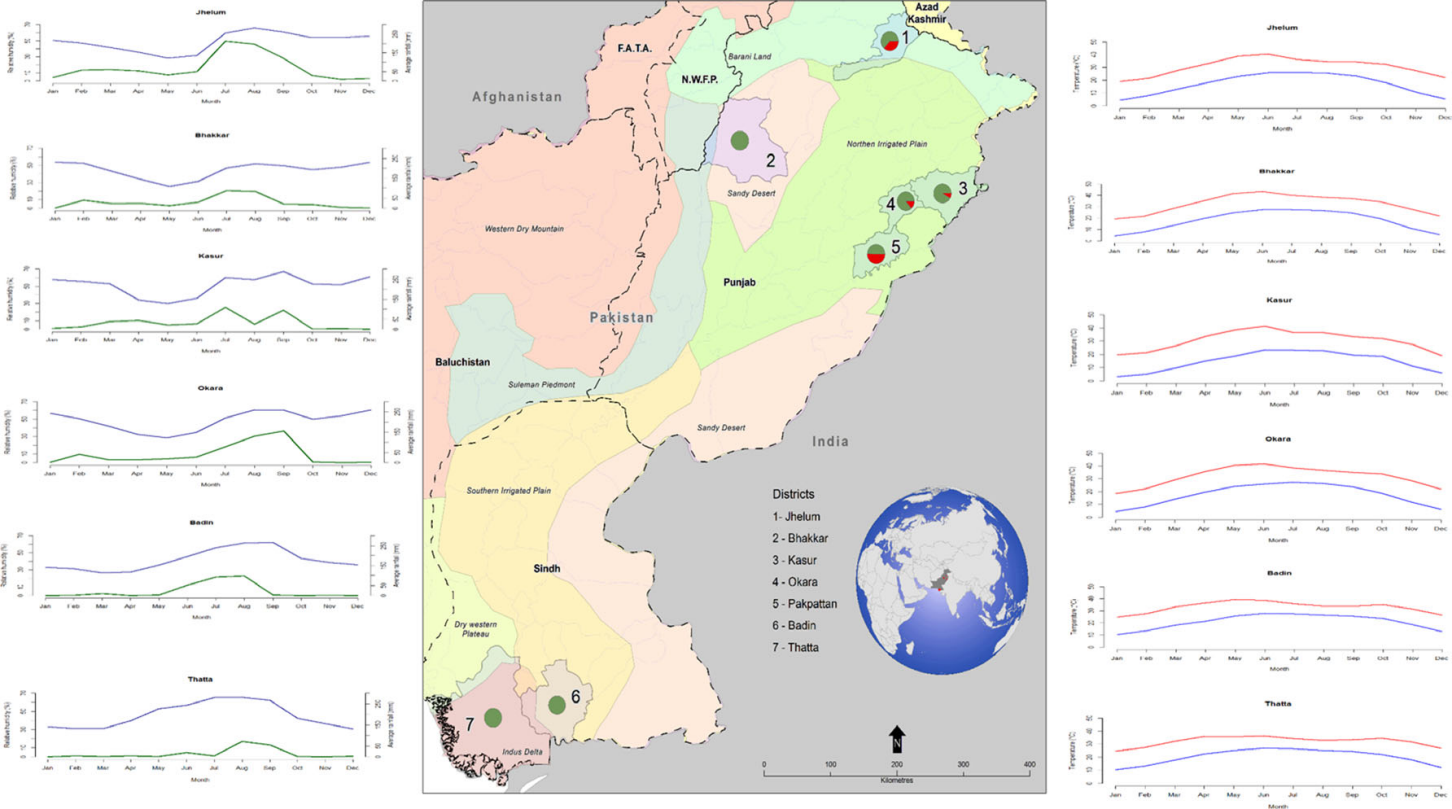
Spatial distribution map of Brucella seropositive herds (n = 418) in seven districts of Pakistan. Brucella positive herds (n = 68) are indicated by the red portion of the pie graphs, whereas negative herds (n = 350) are shown in green. The coloured areas on the map indicate the different agro‐ecological zones of Pakistan. Climate data regarding temperature is plotted on right side (red line for max and blue for min) for six districts. Average relative humidity and rainfall data are plotted on the left side (blue line represents humidity and green for rainfall). The map was created using ArcGIS ^®^ software by Esri. ArcGIS ^®^ and ArcMap™.

Sixty‐eight of the 418 herds had at least one seropositive buffalo or cattle with RBT or C‐ELISA, which resulted in an overall herd‐level prevalence of 16.2% (95% CI, 13–20%). Similarly, 93 out 1063 animals were positive, resulting in an animal‐level seroprevalence of 8.7% (95% CI, 7.15–10.6%) using both of these tests in parallel combination. Figure 1 shows the animal‐level prevalence across the seven districts in buffalo and cattle. A greater proportion of buffalo compared with cattle is present in Pakpattan, Jhelum, Okara and Kasur and also a higher herd and animal‐level prevalence was found in these districts. Districts Bhakkar, Thatta and Badin have no positive animals with either tests. The spatial distribution of *Brucella* seroprevalence is shown in Fig. 2.

### Herd‐level risk factor association with *Brucella* seropositivity

**Figure 3:**
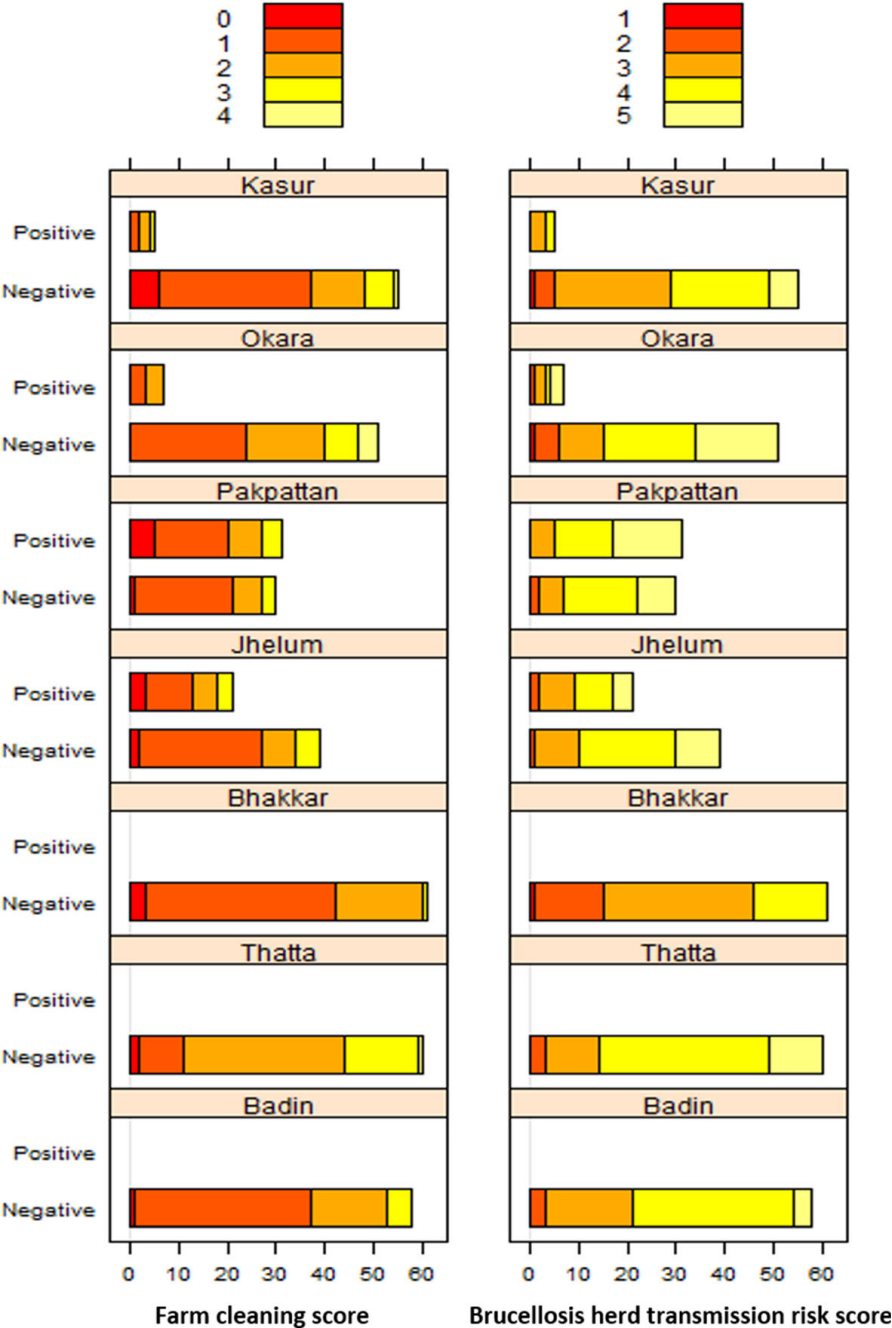
Distribution of farm cleaning score and brucellosis herd transmission risk score among positive herds and negative herds for brucellosis in seven districts of Pakistan. 0–5 indicates the total number of practices undertaken.

The univariable logistic regression models show that three of the six herd‐level risk factors had a significant association with herd positivity, namely, number of buffalo, retained placenta cases in last year and last trimester abortion in last year (all *P *≤* *0.05), whereas the number of cattle (*P *=* *0.88), presence of other domestic animals (*P *=* *0.52) and purchasing animals in last year (*P *=* *0.096) had no significant association. Herds with a greater number of buffalo were associated with disease presence (Table 2). However, it is important to note that there are greater numbers of buffalos in the seropositive districts compared with districts with no positive herds. Similarly, a history of last trimester abortions in the previous year was significantly associated with herd positivity. Results from the final multivariable logistic regression found only the number of buffalo had a significant association with herd positivity (hence equivalent to the univariable results in Table 2). Generally, herds with five or more buffaloes were associated with greater herd positivity compared with herds fewer buffaloes on the farm. The distribution of herd management practices scores among positive and negative herds for bovine brucellosis is illustrated in Fig. 3.

**Table 2:** Summary of univariable models investigating potential explanatory risk factors for herd‐level prevalence with RBT and C‐ELISA in parallel combination as the outcome variable. *P*‐value is shown for each explanatory variable followed by the odds ratio (OR) and 95% confidence interval (95% CI) for the OR

Explanatory variables	Parallel combination of RBT and C‐ELISA	
Number of buffalo	*P* = 0.0026**	
	OR	95% CI
1–2	1	
3–4	2.02	(0.93, 4.38)
5–8	3.80	(1.69, 8.49)
>8	3.81	(1.51, 9.58)
Number of cattle	*P* = 0.88	
1–2	1	
3–4	0.81	(0.35, 1.83)
5–8	0.98	(0.25, 3.83)
Retained placenta cases in last year	*P* = 0.0041**	
No	1	
Yes	1.69	(1.16, 2.46)
Last trimester abortion in last year	*P* = 0.027*	
No	1	
Yes	2.06	(1.09, 3.89)
Presence of other domestic animals at farm	*P* = 0.52	
No	1	
Yes	0.82	(0.46, 1.47)
Animal purchased in last year	*P* = 0.096	
No	1	
Yes	0.61	(0.34, 1.09)

**Table 3:** Summary of univariable models for animal‐level risk factors and animal‐level prevalence with RBT and C‐ELISA in parallel combination as the outcome variable. P‐value is shown for each explanatory variable followed by the odds ratio (OR) and 95% confidence interval (95% CI) for the OR

Animal level risk factors	Parallel combination of RBT and C‐ELISA	
Species	*P* = 0.0018	
	OR	95% CI
Buffalo	1	
Cattle	0.44	(0.25, 0.75)
Age (years)	*P* = 0.28	
1–2	1	
4–6	1.45	(0.80, 2.62)
6–8	1.72	0.93, 3.16)
>8	1.70	(0.83, 3.45)
Body condition score	*P* = 0.71	
1–2	1	
2–3	1.21	(0.64, 2.28)
3–4	1.54	(0.53, 4.46)

## Discussion

This study shows that bovine brucellosis, as defined by seropositivity of either RBT or C‐ELISA, is present in smallholder dairy farms in four districts in Pakistan. The herd‐level and animal‐level prevalence was 16.2% and 8.7%, respectively, on mixed cattle and buffalo farms across four districts. However, no herd or animal was found positive with *Brucella* in Bhakkar, Thatta and Badin districts. To our knowledge, this is the most comprehensive study of bovine brucellosis in terms of smallholder settings. A recent study conducted in Potohar plateau, Pakistan, has reported very similar results, with 18.6% herd‐ and 6.3% animal‐level prevalence with RBT (Ali *et al*. 2017). Another study carried out at veterinary hospitals, animal markets and large peri‐urban farms in Rawalpindi and Islamabad has shown very low animal‐level prevalence, namely 1.6% in buffalo and 6.6% in cattle (Ahmad *et al*. 2017). Studies from other neighbouring countries such as India, Bangladesh, Iran and Sri Lanka have reported a wide range of prevalence estimates, from an overall prevalence of 27% in cattle and buffalo with RBT, 9.7% in cattle with RBT and ELISA, 0.7% in cattle with RBT and SAT (serum agglutination test) and 4.7% in cattle and 4.2% in buffalo with I‐ELISA, respectively (Silva *et al*. 2000; Mombeni *et al*. 2014; Zadon & Sharma 2015; Ahasan *et al*. 2017). These estimates from different countries with different production systems highlight the variability in *Brucella* prevalence across different environments and systems. However, it is important that we attempt to understand some of the variation we might get and also identify the drivers behind the variation in prevalence estimates.

Animal production system as well as environment can greatly influence the spread of bovine brucellosis (WHO, 1997). A review carried out in Ethiopia reported that brucellosis prevalence varies between agro‐ecological zones (Yilma *et al*. 2016). In this study, disease was found in the northern irrigated agro‐ecological zone which is an arid zone by agro‐climatic definition. This study does show that *Brucella* varies between agro‐ecological zones which informs us of the importance to have targeted intervention to the areas where the disease is more likely a concern. As Table 1 shows, there is high average humidity in the districts where *Brucella* is present and it is reported that bacteria survive in humid environments for longer than in colder or drier periods (Rodríguez‐Morales, 2013). Districts where the disease was absent were found to be much drier and the districts where it is present have greater access to rivers/irrigation water. It is reported that climate variables are associated with the disease prevalence and distribution (Li *et al*. 2013; Ahmadkhani & Alesheikh 2017) but further investigation is required to confirm this in Pakistani context, using finer‐level climate data.

This study revealed that last trimester abortion, the number of buffalo on the farm, and history of retained placenta in the herd were positively associated with herd‐level prevalence. The association with last trimester abortion is in agreement with the biology of *Brucella* (McDermott *et al*. 1987) and similar findings have been reported in other studies (Boukary *et al*. 2013; Lindahl *et al*. 2014). In addition, the association between herd size and presence of *Brucella* has been demonstrated on smallholder farms previously. For example a study conducted in Ethiopia found that higher seroprevalence in cattle was observed in larger‐sized herds (Ibrahim *et al*. 2010). This study was conducted on smallholder farms having a maximum of 10 animals, mostly mixed cattle and buffalo farms. This study found that the odds of a herd being seropositive for brucellosis was higher when there were five or more buffaloes in the herd than when there were less than five. These findings possibly suggest that increased numbers of buffaloes on farm may be a risk factor for bovine brucellosis. However, these results may also reflect the greater number of buffalo compared with cattle in the disease‐positive districts, which may be independent of the previous putative association (see Table 2). Note the sampling approach was consistent across all districts, so it is less likely to introduce bias. No association was found between *Brucella* and the presence of other domestic animals on farm, nor with history of new animals being purchased in the past year, although some other studies have reported an association with purchasing of new animals (Matope *et al*. 2010). Notably, in Pakistan, smallholder farmers have no record keeping system and unrestricted movements that were not able to be documented in this study could be a contributing factor to disease presence and spread.

Two herd management practices scores were used in this study, as described earlier, namely farm cleaning practices score and brucellosis herd transmission risk score. The distribution of herd management practices among positive and negative farms displayed (Fig. 3) shows that the majority of farms were carrying out at almost all risky practices in all districts, whereas some districts (Pakpattan and Jhelum) have higher disease and higher brucellosis risk transmission scores. In general, farms have better cleaning scores than brucellosis risk transmission scores. There is room for improvement in the herd management practices across all the districts. While there was no association between these scores and presence or absence of the disease in the districts, it is likely that they contribute where the disease exists. Therefore, targeted intervention may be useful in either reducing spread of disease or preventing spread of disease if it introduced to that environment. These practices are traditionally present there and embedded in the culture so intervention is also required to be applied at wider level than individuals. Indeed, high levels of risky practices were reported previously (Arif *et al*. 2017).

At the animal level, there were significant differences in seropositivity between the two species, with buffalo being more likely to be seropositive for brucellosis than cattle. This is also consistent with the herd‐level analysis, where associations were found with buffalo herd numbers but not cattle numbers. Age and body condition score were found to have no association with disease presence at the animal level. However, animal age has previously been reported as a risk factor (Matope *et al*. 2010). As the overall body condition score in smallholder farms is very poor, with an average of less than three and little variation, it is unlikely that we would find an association with *Brucella* in this environment. Although animal sex was not investigated in this study as there was low availability of males, presence of infected male animals on farm has previously been identified as a risk factor for the disease spread (Bayemi *et al*. 2009).

In this study cattle and buffalo sampling was based on availability in general, as smallholders have few animals, mostly as mixed farms and in some areas there are more buffalos than cattle. While this may have introduced sampling bias it reflects the distribution of animals in the regions sampled. Another potential bias could be recall bias as the farmers were asked to recall the event one year before, however, this is commonly practiced in cross‐sectional studies.

In conclusion, this study has identified herd‐level and animal‐level risk factors associated with seropositivity for brucellosis. Given there is presence of risky practices across all districts and four districts with high prevalence of the disease, region/district‐specific targeted interventions are required, with those areas with high prevalence prioritised initially. While the districts with high disease prevalence should be prioritised and programmes with disease control measures and educational campaigns should be developed and implemented, the districts with no or very low prevalence of disease, although being considered a lower priority for the disease, should not be omitted and educational awareness programmes focusing on preventive measures, should also be implemented. The findings of this study could also be helpful to understand the risk factors for bovine brucellosis for other neighbouring countries with similar smallholder settings.

## Source of funding

Author (SA)'PhD was supported by ACIAR (Australian Centre for International Agricultural Research) through PhD scholarship. Field and laboratory work for this study supported by ASLP Dairy Project in Pakistan which is also funded through ACIAR.

## Conflict of interest

The authors declare that they have no conflict of interest.

## Ethics statement

The study was approved by the Animal Care and Ethics Committee, and Human Research Ethics Committee of Charles Sturt University Australia (Approval Numbers 15/003, 2014/222), in conjunction with staff at University of Veterinary and Animal Sciences, Lahore Pakistan.

## Contributions

SA JH MHJ PT DM, Data curation: SA KH HMW, Formal analysis: SA PT JH MHJ, Methodology: SA JH PT MHJ DM, Project administration: SA, Resources: SA DM KH, Supervision: JH PT MHJ, Writing‐ original draft: SA, Writing‐review & editing: JH PT MHJ DM.
